# Autoimmune hepatitis related autoantibodies in children with type 1 diabetes

**DOI:** 10.1186/1758-5996-6-38

**Published:** 2014-03-17

**Authors:** Abdulrahman A Al-Hussaini, Musa D Alzahrani, Ahmed S Alenizi, Nimer M Suliman, Mannan A Khan, Sahar A Alharbi, Aziz A Chentoufi

**Affiliations:** 1 Department of Pediatrics at King Fahad Medical City, Riyadh, Saudi Arabia; 2 Department of Pediatrics at King Saud Medical City, Riyadh, Saudi Arabia; 3 Department of Radiology at King Saud Medical City, PO box 7855, Riyadh 11117, Kingdom of Saudi Arabia; 4 Department of Immunology, Pathology, Clinical Laboratory Medicine at King Fahad Medical City, Riyadh Saudi Arabia; 5 Division of Pediatric Gastroenterology, Hepatology & Nutrition, University of King Saud Bin Abdulaziz for Health Sciences, Children's Hospital, King Fahad Medical City, Riyadh, Saudi Arabia; 6 Division of Pediatric Endocrinology, Children's Hospital, King Saud Medical City, PO box 7855, Riyadh 11117, Kingdom of Saudi Arabia

**Keywords:** Type 1 diabetes, Liver autoantibodies, Antinuclear antibody, Smooth muscle antibody, Anti-liver kidney microsomal antibody

## Abstract

**Background and objectives:**

The frequency of Type 1 diabetes (T1D)-related autoantibodies was determined in children with autoimmune hepatitis. However, the incidence of autoimmune hepatitis related autoantibodies in children with T1D has been poorly investigated. The aim of the present cross sectional prospective study was to determine the occurrence of autoimmune hepatitis-related autoantibodies in children with T1D.

**Methods:**

Children with T1D following in diabetic clinic in our center were screened for existence of liver related autoantibodies from November 2010 to November 2011. The patients’ sera were analyzed for the existence of autoantibodies such as anti-nuclear antibody, anti-smooth muscle antibody, and anti-Liver Kidney microsomal antibody, using enzyme linked immunoassay and indirect immunofluorescence methods. A titer of anti-nuclear antibody ≥1/40 was considered positive and titer of < 1/40 was considered negative. Anti-liver kidney microsomal antibody titer of < 3 U/ml was considered negative, 3 – 5 U/ml borderlines, and > 5 U/ml was considered positive.

**Results:**

106 children with T1D have been examined over a one-year period: age ranges between 8 months to 15.5 years, sixty two patients were females. Autoantibody screen revealed a girl with positive anti-liver kidney microsomal antibody (1%) and 8 children had positive anti-nuclear antibody (7.5%), without clinical, biochemical or radiologic evidence of liver disease. None of the patients had positive smooth muscle antibody.

**In conclusion:**

Anti-liver kidney microsomal antibody is rarely found in sera of children with T1D; the clinical significance of which is unknown.

## Introduction

Type 1 diabetes (T1D) is a disorder of glucose metabolism that results from insulin deficiency secondary to autoimmune destruction of insulin – secreting β cells. The finding of organ–specific autoantibodies in patients with T1D discloses the existence of a wide spectrum of immunological diseases. Fifteen to 30% of patients with T1D have autoimmune thyroiditis, 4-9% have celiac disease, and 0.5% have Addison’s disease [1]. Autoimmune hepatitis (AIH) has been reported to occur in patients with T1D [2-7]. The frequency of T1D-related autoantibodies was determined in children with AIH: islet cell antibodies and insulin autoantibodies were found in 60.7% and 18.5% of patients with AIH, respectively [4]. However, the co-occurrence of AIH and the frequency of AIH type 1 related auto-antibodies, such as anti-nuclear antibody (ANA) and anti-smooth muscle antibodies (SMA), and anti-Liver/Kidney microsomal (LKM-1) antibodies in AIH type 2, in patients with T1D has only been recently investigated in very limited number of studies [8,9]. In a retrospective study, Allen at al. had screened adults with T1D for presence of organ specific autoantibodies; 2 out of 261 (0.8%) were positive for SMA and none was positive for LKM-1 antibody [8]. The serologic tests used in this study to detect SMA and LKM-1 antibodies were not mentioned. In a controlled study in adults, Heras et al. found that19 of 70 patients (27%) with T1D had positive ANA, as compared to 4 of 28 patients with type 2 diabetes (14%) and 4 of 20 healthy controls (20%) [P < 0.001] [9].

Our cross sectional prospective study is the first to report on frequency of AIH related autoantibodies in children with T1D.

### Patients and methods

Children with T1D in follow up in diabetes clinic in our tertiary care center were consecutively enrolled into the study over the period from November 2010 to November 2011. Type 1 diabetes was diagnosed by the onset of hyperglycemia, the absolute dependence on insulin treatment for survival, and by the presence of severe deficiency of insulin secretion (i.e., very low or undetectable fasting C–peptide levels) and of anti-islet cell auto-antibodies. We excluded children with other disease that might primarily affect the liver. The study has been conducted after parental consent. The following have been collected: age, sex, age of onset of T1D, duration of T1D, and individual and family history of autoimmune diseases. Blood has been collected and the sera were used for the examination of the presence or absence of the following autoantibodies:

#### A- Anti-nuclear antibodies screening

Anti-Nuclear Antibodies were detected using DiaSorin kits Immunofluorescence assay (DiaSorin, Saluggia, Italy), Serum samples were diluted to 1:40 then added for 30 minutes to the Hep2 cells fixed on the slides. After washing, the FITC-labeled conjugate was added for 30 min incubation. After washing step, the mounting media was added and slides were cover slipped and analyzed for nuclear patterns using florescent microscope. The observation of positive staining with specific pattern on the substrate indicates the presence of ANA in the test sample. A titer of ANA ≥1/40 was considered positive and titer of < 1/40 was considered negative. To rule out systemic lupus erythematosus (SLE), all positive samples were screened for double-stranded DNA (dsDNA) using LIAISON dsDNA chemiluminescence assay (DiaSorin, Saluggia, Italy).

#### B- Anti-smooth muscle antibodies (SMA)

Anti-Smooth Muscle Antibodies were detected using solid phase enzyme immunoassays using INOVA Diagnostics (San Diego, CA) ELISA kit. Antigens coated 96 wells plates were incubated for 30 minutes with calibrators, positive and negative controls and diluted serum sample specimens. During incubation, antibody present in the test sample binds to the coated well. Following the 30 minutes incubation, the wells were washed and horseradish peroxidase-conjugate anti-human IgG was added to the wells. Then the substrate was added for 30 minutes and the reaction stopped. The optical density was measured by spectrophotometer. The cutoff was <20 U/IU and whenever the result was positive, we performed F-actin IgG enzyme linked immunoassay kit from INOVA Diagnostics (San Diego, CA) as confirmatory test.

Alternatively, anti-SMA screening was done by Immunofluorescence assay using INOVA Diagnostics (San Diego, CA) kit, serum sample was diluted to 1:20 then added on a slide and incubated for 30 minutes. After washing, the FITC-labeled conjugate was added for 30 min incubation. After 10 min washing step, the mounting media was added and the slides were cover slipped and viewed for fluorescent patterns under florescent microscope. Observation of specific patterns on the substrate indicates the presence of anti-SMA in the test sample. A titer of Anti-SMA > 1/20 was considered positive and titer of < 1/20 was considered negative. Any time the results were positive, a confirmatory assay was done using anti-F-actin autoantibodies screening using enzyme linked immunoassay kit from INOVA Diagnostics (San Diego, CA).

#### C- Anti-liver/kidney microsome (LKM-1) antibodies

Anti-Liver/Kidney Microsome antibofies were detected using ELISA kit from DiaSorin (DiaSorin, Saluggia, Italy). LKM-1 Antibodies were detected by solid phase enzyme immunoassays. Antigen precoated 96 well plates were incubated with calibrators, controls and serum specimens. Then, horseradish peroxidase-conjugated anti-human IgG was added to the wells. The substrate was added, the reaction stopped and auto-antibodies were measured using a spectrophotometer plate reader. LKM-1 antibody titer of < 3 U/ml was considered negative, 3 – 5 U/ml borderline, and > 5 U/ml was considered positive. Positivity for LKM-1 antibody was an indication to screen for anti-HCV antibody.

Cu-off values for the detection of the autoantibodies in this study were used as recommended by the manufacturers and validated by serology laboratory. Children who tested positive for any of the above autoantibodies have undergone further evaluation for the presence of liver disease including: physical examination for hepatomegaly (by measuring the distance between the upper and lower borders of the liver at mid-clavicular line using a tape measure), blood tests for liver function tests, total serum immunoglobulin, complement C3 and C4, and ultrasound of liver. The length of the liver was measured from uppermost portion of the dome of diaphragm to the inferior tip. Length of liver above standard deviation (SD) of mean for normal liver size in children [10] was reported as hepatomegaly. Uniform pattern of medium strength echoes from liver was considered as normal whereas increased strength of echoes resulting into marked liver-kidney echogenic discrepancy and poor definition of hepatic veins, portal vein, and crus of diaphragm was considered as increased echogenicity of liver. Liver biopsy was indicated if there is any of the following: hepatomegaly, hyperechogenic liver on ultrasound or elevation of ALT (normal, 30–65 IU/L) and AST (normal, 15–37 IU/L) > 1.2 times of normal values. Patients with positive AIH–related auto-antibodies but normal ALT and AST and no abnormal finding on ultrasound were not candidates for liver biopsy but were followed up regularly in the clinic with repeated functional liver tests and autoantibody every 6 months.

The study was approved by the review board at King Saud Medical City and had been performed in accordance with the ethical standards laid down in the 1964 Declaration of Helsinki and its later amendments.

## Results

**Figure 1:**
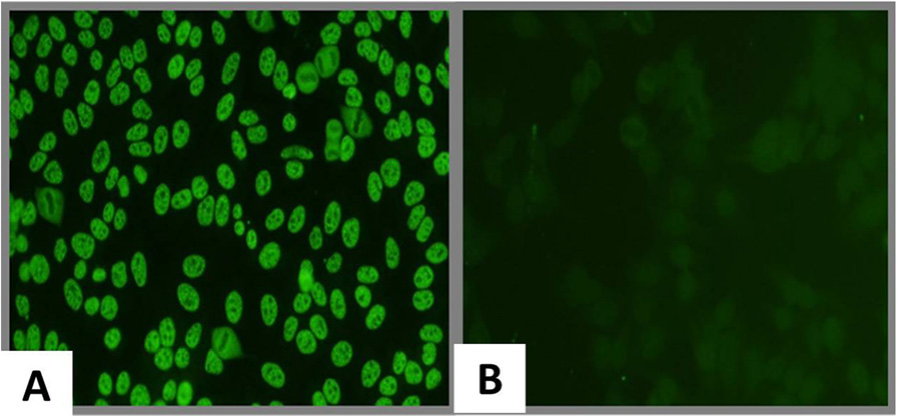
**Immunofluorescence staining. (A)** Speckled immunofluorescence pattern of anti-nuclear antibody, **(B)** Healthy control.

Over the one-year study period, 107 children with T1D were identified. One of them has β-thalasemia major and hepatomegaly so was excluded from the study; 106 children with T1D were investigated. The demographic, clinical, and biochemical characteristics of the 106 children are shown in [Table 1]. Seven patients were newly diagnosed with T1D. Screening of diabetic children for presence of AIH-related autoimmune markers revealed the following: 8 had high ANA titer (7.5%), and one was positive for LKM-1 antibody (1%) [Table 2]. All of the ANA positive patients were negative for dsDNA. None of these patients had hepatomegaly, hyperechogenic liver on ultrasound or elevation of ALT and AST > 1.2 times of normal values; a criterion we have set in our study protocol to justify doing liver biopsy. All of the patients had normal serum immunoglobulin G. All ANA positive patients demonstrated speckled immunofluorescence pattern (Figure 1). Only patient 1 has concomitant autoimmune disease with positive anti-tissue transglutaminase antibody of 109 U/ml and biopsy proven celiac disease.

**Table 1:** Clinical and laboratory characteristics of 106 children with T1D

	**Patients (n = 106)**
	**Mean ± SD**
**Age (year)**	8.5 ± 2.8
**Gender (female/male)**	62/44
**Age at diagnosis of T1D (year)**	6.3 ± 2.9
**Duration of diabetes (year)**	2.2 ± 2.1
**Body mass index (kg/m**^**2**^**)**	16.5 ± 3.4
**HbA1c (%)**	10.7 ± 2.4
**ALT (U/L)** (normal, 30–65 IU/L)	28 ± 8.3
**AST (U/L)** (normal, 15–37 IU/L)	25.7 ± 10.4
**Serum cholesterol** (normal 3.65–5.15 mmol/L)	4.16 ± 0.75
**Serum triglyceride** (normal 0–1.7 mmol/L)	1.02 ± 1.3

ALT = Alanine aminotransferase; AST = Aspartate aminotransferase; HbA1c = Glycosylated hemoglobin; T1D = Type 1 diabetes.

**Table 2:** Patients with T1D and positive autoantibodies

**Patient**	**Age (year)**	**Sex**	**Positive autoantibody**	**Associated auto-immune disease**
1	4	F	ANA 1/640	Celiac disease
2	6.5	F	ANA 1/160	None
3	11	F	ANA 1/80	None
4	6	F	ANA 1/640	None
5	15	M	ANA 1/80	None
6	7.5	M	ANA 1/320	None
7	9	M	ANA 1/80	None
8	7.5	F	ANA 1/80	None
9	6	F	^a^Anti-LKm 6.8 U/ml	None

T1D: Type 1 diabetes; ANA: Anti-nuclear antibody; Anti-LKm: Anti-liver kidney microsomal antibody; F: Female, M: Male; ^a^normal anti-LKM1 = 3–5 U/ml.

Two patients initially had a weakly positive SMA but both showed negative results on repetition after 6 months. The patient with positive LKM-1 antibody was non-reactive to anti-HCV antibody, and repeat of the test in 6, 12, and 18 months demonstrated a persistently positive LKM-1 antibody.

## Discussion

The major finding of our study is the rare finding of positive LKM-1 antibodies in one child out of 106 children with T1D (1%). Eight more patients have been identified to have high titers of ANA.

Patients with T1D show a high prevalence of autoimmune aggression against organs like thyroid gland, small bowel, adrenal gland, and gastric mucosa which documents the autoimmune dysregulation in such patients. Fifteen to 30% of patients with T1D have autoimmune thyroiditis, 4-9% have celiac disease, 0.5% have Addison’s disease, [1] and 20% have parietal cell antibodies [11]. The presence of organ specific autoantibodies (thyroid peroxidase and thyroglobulin antibodies with autoimmune thyroiditis, EMA and TTG autoantibodies with celiac disease, and 21-hydroxylase autoantibodies with Addison disease) can precede the development of overt disease, [1] therefore these organ specific autoantibodies can provide a simple way to screen for autoimmunity and can predict the development of autoimmune disease in a susceptible population.

Autoimmune liver disease, particularly AIH type 2, has been reported to develop in patients with T1D, and vice versa [3-7]. The frequency of T1D-related autoantibodies was determined in 28 children with AIH: islet cell antibodies and insulin autoantibodies were found in 60.7% and 18.5% of patients, respectively; [4] none of these patients developed T1D after 3 – 9 years of follow up. Anti-glutamic acid decarboxylase antibodies were present in only one patient who developed T1D after 3 years [5]. On the other hand, the frequency of AIH related auto-antibodies (ANA and SMA in AIH type 1, and Anti-LKm in AIH type 2) in patients with T1D has been poorly investigated in adults [8,9] and data are lacking in children. LKM1 autoantibodies are directed against cytochrome P450 2D6 (CYP2D6), an enzyme expressed by hepatocytes [12]. Carboxypeptidase H (CPH), a candidate molecular target in T1D, share sequence similarity to the second major LKM epitope on CYP2D6. The second major epitope of CYP2D6 (CYP2D6_321–351_) shares a common amino acid motif with CPH_33–51_[3]. This data suggest that autoimmunity to a tissue specific autoantigen may involve other organs, expressing proteins that share sequence similarity to the initial autoantigen, by a cross reactive immune mechanism, and may lead to clinically apparent autoimmune disease in some susceptible individuals. Homologies also exist between CYP2D6 and the genome of HCV, and cross reactivity may account for anti-LKm in as many as 10% of patients with HCV infection [13]. Our study identified positive Anti-LKm antibodies in a 6 year-old girl with T1D (1%), in absence of clinical or biochemical evidence of overt liver disease. This finding may be the result of molecular mimicry between CPH in T1D and the target of LKm-1 antibody (CYP2D6), leading to a cross reactive immune response. The main target in AIH type 2 is an amino acid region on CYP2D6 that lies between peptides 257–269; [14] immunreactivity associated with HCV is usually directed against diverse epitopes of CYP2D6 [11]. In T1D, autoantibodies to islet cell components are present many years before the onset of overt disease and are associated with higher risk of developing T1D [15]. Whether our patient with T1D, but reactive to LKM-1 antibodies, will eventually develop overt AIH type 2 remains to be seen. Screening for type of CYP2D6 epitopes, a procedure that we can not do in our laboratory, can distinguish the antibody subtypes and may help verifying the clinical significance of the antibody detected. Long term, prospective larger studies are needed to identify the natural history of autoimmunity against the liver in patients with T1D.

ANA and SMA are clues for the diagnosis of AIH type 1. They may be consequences of liver injury rather than the cause. They typically lack disease specificity, fluctuate in titer, and variably appear and disappear in individual patients [16]. Eight of our patients with T1D demonstrated high ANA titers without SMA positivity or a clinical or biochemical evidence of liver dysfunction. Thirteen percent of patients with AIH type 1 have only ANA at presentation and SMA develop later in 50% of patients [17]. Interpretation of the significance of ANA reactivity in our patients should be tempered by the fact that ANA reactivity, albeit in low titer, is commonly seen in sera from normal individuals [18]. The presence of autoantibodies, regardless of the titer, does not establish a diagnosis or justify treatment if not supported by other findings [17]. It is possible that autoimmune dysfunction in patients with T1D could have contributed to ANA reactivity, as systemic non-liver autoimmune diseases, like systemic lupus erythematosus, rheumatoid arthritis, Sjogren’s syndrome or other connective tissue diseases, are a well-recognized causes of ANA positivity [19]. However, it seems unlikely that any of the ANA-positive patients in this study have sub-clinical systemic lupus erythematosus as all were anti-double stranded DNA negative. Long-term follow-up of the entire autoimmune spectrum of ANA positive patients, especially those with titres >1/320 is required. This will allow us to determine whether the antibodies are transient findings, following any epigenetic antigenic stimulus or a precursor of an autoimmune systemic disease of pathogenic prognostic significance. Larger confirmatory studies are required to further evaluate the findings of this study.

An important limitation of our study is not testing for other serological markers of autoimmune hepatitis: anti-soluble liver antigen and anti-liver cytosol type 1 [20]. Therefore, future studies should consider testing for complete panel of AIH-related autoantibodies. Another limitation is the small sample size and lack of a control group to help understand how specific our findings to T1D.

## In conclusion

Reactivity to anti-LKm1 and ANA in sera of patients with T1D is rare and could be due to the autoimmune dysfunction characterizing patients with T1D. The clinical significance of these autoantibodies in children with T1D is yet to be identified in a long term larger prospective studies.

## Abbreviations

AIH: Autoimmune hepatitis; ALT: Alanine aminotransferase; ANA: Anti-nuclear antibody; LKM-1: Liver/kidney type 1 microsomal antibody AST: Aspartate aminotransferase; BMI: Body mass index; CPH: Carboxypeptidase H; HbA1C: Glycosylated hemoglobin; HCV: Hepatitis C virus; SMA: Smooth muscle antibodies; T1D: Type 1 diabetes.

## Competing interests

Authors disclose no conflict of interest and have no financial relationships to disclose. We acknowledge that the sponsor of this research “King Abdulaziz City for Science and Technology” (KACST) thru a grant No. LPG-10-41. KACST had no role in the study design, data collection or analysis, manuscript writing, or submission for publication.

## Authors’ contributions

ARH is the principal investigator that designed, conducted the study and interpreted the data, and wrote the draft of this manuscript; MZ collated and assisted in the interpreted the data, and assisted in writing the manuscript; AA collated and assisted in the interpreted the data, and assisted in writing the manuscript; NS provided services associated with the design, conduct of the study, the interpretation of the data, and assisted in writing this manuscript. SA is immunology laboratory technician who has performed the serology tests, and assisted in writing the methodology. AC has contributed to study design, conduct of the study, interpretation of serology tests, and assisted in writing the manuscript. All authors read and approved the final manuscript.
